# Developing a learning health system: Insights from a qualitative process evaluation of a pharmacist-led electronic audit and feedback intervention to improve medication safety in primary care

**DOI:** 10.1371/journal.pone.0205419

**Published:** 2018-10-26

**Authors:** Mark Jeffries, Richard N. Keers, Denham L. Phipps, Richard Williams, Benjamin Brown, Anthony J. Avery, Niels Peek, Darren M. Ashcroft

**Affiliations:** 1 Centre for Pharmacoepidemiology and Drug Safety, Division of Pharmacy and Optometry, School of Health Sciences, University of Manchester, Manchester, United Kingdom; 2 NIHR Greater Manchester Patient Safety Translational Research Centre, University of Manchester, Manchester Academic Health Sciences Centre (MAHSC), Manchester, United Kingdom; 3 Health eResearch Centre, School of Health Sciences, The University of Manchester, Manchester, United Kingdom; 4 Division of Primary Care, University of Nottingham, Nottingham, United Kingdom; Newcastle University, UNITED KINGDOM

## Abstract

**Introduction:**

Developments in information technology offer opportunities to enhance medication safety in primary care. We evaluated the implementation and adoption of a complex pharmacist-led intervention involving the use of an electronic audit and feedback surveillance dashboard to identify patients potentially at risk of hazardous prescribing or monitoring of medicines in general practices. The intervention aimed to create a rapid learning health system for medication safety in primary care. This study aimed to explore how the intervention was implemented, adopted and embedded into practice using a qualitative process evaluation.

**Methods:**

Twenty two participants were purposively recruited from eighteen out of forty-three general practices receiving the intervention as well as clinical commissioning group staff across Salford UK, which reflected the range of contexts in which the intervention was implemented. Interviews explored how pharmacists and GP staff implemented the intervention and how this affected care practice. Data analysis was thematic with emerging themes developed into coding frameworks based on Normalisation Process Theory (NPT).

**Results:**

Engagement with the dashboard involved a process of sense-making in which pharmacists considered it added value to their work. The intervention helped to build respect, improve trust and develop relationships between pharmacists and GPs. Collaboration and communication between pharmacists and clinicians was primarily initiated by pharmacists and was important for establishing the intervention. The intervention operated as a rapid learning health system as it allowed for the evidence in the dashboard to be translated into changes in work practices and into transformations in care.

**Conclusions:**

Our study highlighted the importance of the combined use of information technology and the role of pharmacists working in general practice settings. Medicine optimisation activities in primary care may be enhanced by the implementation of a pharmacist-led electronic audit and feedback system. This intervention established a rapid learning health system that swiftly translated data from electronic health records into changes in practice to improve patient care. Using NPT provided valuable insights into the ways in which developing relationships, collaborations and communication between health professionals could lead to the implementation, adoption and sustainability of the intervention.

## Introduction

Prescribing medication is one of the most common processes in primary healthcare, with over one billion prescription items issued each year in England alone.[1, 2] Unlike much of Europe and the USA the first contact for patients in the UK is almost always with a GP rather than a specialist and therefore much acute and repeat prescribing takes place in primary care. In the UK general practices are self-contained organisations of GPs which might employ nurses and pharmacists to help them with prescribing and/or manage medicines optimisation. Medicines optimisation has been defined as 'a person‑centred approach to safe and effective medicines use, to ensure people obtain the best possible outcomes from their medicines’.[3] Within primary care it has been shown that errors can occur in the prescribing of medicines or where patients in receipt of prescribed medicines are not routinely monitored through routine blood tests.[4, 5] In a retrospective study of prescribing in 15 English general practices over one year, the prevalence of monitoring or prescribing errors in over 6000 unique prescription items was 4.9%, with 18.7% of patients receiving at least one prescribing or monitoring error.[4] Between 5% and 13% of hospital admissions are due to adverse drug events (ADE) following patients receiving prescription medication in primary care.[6, 7] Similarly in primary care in the USA it has been found that 25% of patients had an ADE after receipt of at least one prescription in a four week period, with 13% of these deemed serious and 11% preventable. [8] Finding ways to routinely monitor prescribing safety in order to avert potential harm is therefore key.[4] Prescribing safety indicators describe prescribing and monitoring events that may place patients at risk of harm: a recent study using electronic primary care health record data from across the UK reported that 5.26% (95% CI 5.21% to 5.30%) of patients were affected by at least one prescribing safety indicator and 11.8% (11.6% to 11.9%) were affected by at least one monitoring indicator.[9] In the English city of Salford, data from general practices revealed similar rates of 5.45% for prescribing and 7.65% for monitoring indicators, respectively.[10]

The increasing use of information technology (IT) in healthcare offers new opportunities to enhance medication safety.[11, 12] However, implementation of IT-based interventions has previously encountered problems through limited user acceptance, issues with ownership and problems embedding the intervention into existing work practices.[13–18] In primary care, IT systems can interrogate population-based prescription records and thus provide surveillance for hazardous prescribing in the community.[10] A recent systematic review of 10 randomised controlled trials of IT interventions to improve medication safety in primary care found that half the studies reported a reduction of medication errors.[19] An example is the pharmacist-led information technology intervention for medication errors (PINCER), which has been shown to be an effective method for reducing a range of clinically important and commonly made medication errors in primary care.[20] At 6-months follow-up, patients exposed to the PINCER intervention had significantly fewer prescribing errors than those in the control group receiving simple feedback on hazardous prescribing. A nested qualitative evaluation of the same trial identified important factors, such as relationship building between pharmacists and GPs, that could underpin successful implementation of the intervention in general practice.[21] It was suggested that future research might explore the opportunities for enhanced integration of pharmacists into the general practice health care team.[22] The intervention described below, builds upon these recommendations but additionally utilises a near-real time surveillance dashboard to create a learning health system to improve medication safety.

The concept of rapid learning health system (LHS) was proposed as a way of gathering new evidence from routine care and deploying that evidence swiftly into practice using cycles of continuous learning and improvement.[22, 23] A LHS utilises IT, such as electronic health records, for real time learning in daily medical practice and the subsequent transformation of care.[22–24] An important aspect of rapid learning health systems is the cyclical process from data to learning to providing feedback to clinicians.[25] By utilising clinical pharmacy support alongside a real-time surveillance dashboard the present intervention has the potential to create a rapid learning health system for medication safety in primary care.

### The intervention

The intervention was implemented in general practices across the city of Salford, UK, a large urban area in the Northwest of England with a population of 270,000 that is part of the Greater Manchester conurbation. Salford has one hospital, 45 primary care practices, and a shared electronic health record infrastructure between them. The intervention was a complex pharmacist-led intervention designed to identify and feedback instances of potentially hazardous prescribing. Whilst this was based on the PINCER intervention it had two important differences; the access to a real-time surveillance dashboard for pharmacist and GP staff on a day-to-day basis and that pharmacists were based in the participating practices.[26] The dashboard uses a suite of thirteen prescribing and monitoring safety indicators focusing on situations of potentially hazardous prescribing that could result in patients experiencing harm (S1 Appendix). Pharmacists based in the participating general practices identified patients at potential risk of hazardous prescribing and drug monitoring using the dashboard and undertook reviews of their medications, tests and diagnoses using the patient’s electronic medical health records The intervention allowed for all levels of review from medicines use reviews to full clinical medication review with or without the patient present depending on the nature of the hazardous prescribing and the intervention that the pharmacist adopted to best address this. They then worked with the general practice staff to resolve the risk if present. The pharmacists received training in the underpinning evidence-base relating to the safety indicators, use of the dashboard, and root cause analysis informed by the earlier PINCER study.[27] Some pharmacists had previously worked in general practice but others had worked previously in hospital settings. Salford Royal NHS Foundation Trust implemented a Neighbourhood Integrated Practice Pharmacist (NIPP) service in 2016 in which clinical pharmacists were employed to work in general practices to optimise medicines use. A number of these pharmacists were involved in the intervention.

The Medical Research Council has suggested that evaluations of complex interventions need to identify the mechanisms associated with how and why the intervention might lead to outcomes and identify contextual factors.[28, 29] IT interventions to improve health care have been understood as complex programmes delivered in complex settings involving complex processes.[30] Process evaluations need to take into account how the technology is implemented in dynamic organisational contexts.[31–33] A recent systematic review of electronic audit and feedback interventions suggested that insufficient clarity in previous studies meant the active components of interventions could not be fully understood, and recommended that future evaluations of systems should utilise theoretical approaches.[34] This study specifically aimed to explore the ways in which the intervention was implemented, adopted and embedded into everyday practice (or not) using a qualitative process evaluation consisting of semi-structured interviews with pharmacists, general practitioners, and other general practice staff.

## Methods

### Framework: Normalization process theory

Normalization Process Theory (NPT) [35–38] can highlight how an intervention is integrated and adopted into routine practice. NPT has previously been used to evaluate prescribing safety interventions in primary care [39–40], and has utility in examining how individuals and groups understand the intervention through processes of sense-making and how they work to enable the intervention to happen.[35–38] Interventions become part of everyday practice only through the work that people, individually or in groups, undertake.[38] NPT is built upon four constructs: coherence, cognitive participation, collective action and reflexive monitoring [35–38] as described in Table 1.

**Table 1:** Normalization Process Theory (NPT) constructs [35–38].

NPT constructs	
**Coherence**	**Cognitive participation**
The ways in which people define the intervention and how they make sense of it. The work people do to understand how the intervention might involve a different set of practices. The work people do together to understand the intervention and to integrate it into a healthcare setting. The individual tasks involved in the intervention and what people do to attribute worth to that new practice.	How people organise themselves and others through relational work to implement the new intervention. Working together to collectively contribute to the new ways of working that the intervention requires. How the actions and procedures that will be needed to sustain the intervention are defined.
**Collective action**	**Reflexive monitoring**
How the intervention is operationalised and enacted in practice. The collective and interactional work that people do with each other in order to adopt the new intervention into practice. The knowledge work to build confidence in the new practice, divisions of labour and allocation of work including the tasks people do and how those tasks are related to their existing skill sets.	How other individuals and groups evaluate the intervention and look to sustain it. The work that participants do individually and collectively to evaluate and determine how effective the intervention is for them and for others. The impact of the new practices upon their own work. This may include attempts to modify the intervention.

### Recruitment to the intervention

The qualitative process evaluation was carried out during the implementation of the intervention in forty-three general practices across Salford Clinical Commissioning Group (CCG), which serves a population of approximately 270,000 residents. Each individual general practice in Salford CCG was eligible to participate in the intervention. Practice managers initially gave written consent to receive the intervention and participate in the evaluation.

### Recruitment of participants

The study design was a qualitative process evaluation using semi-structured interviews. The sampling frame was determined by the scope of the intervention and therefore included GP staff and pharmacists in practices where the intervention was introduced, and CCG staff who had responsibilities for medicines optimisation and quality improvement in the region. Twenty–eight members of the sampling frame (nine GPs, twelve pharmacists, seven other GP staff) at twenty-three general practices out of the forty-three practices receiving the intervention were directly contacted by MJ via telephone or email and invited to take part in an interview. These practices were purposefully sampled to reflect a range of different contexts and settings which may have had an impact upon how the intervention was implemented and adopted. These included, variations in practice size, social deprivation and clinical systems used (EMIS Web; InPractice systems VISION). Twenty-two participants were recruited from eighteen of these practices. Additionally five CCG managers were approached. Participant roles are detailed in Table 2.

**Table 2:** Details of study participants.

Participant	Practice	Role	Role within Intervention
CCG Manager1	n/a	CCG Quality and Improvement Manager	Aligning intervention with other quality and improvement initiatives
CCG Manager2	n/a	CCG Quality and Improvement Manager	Aligning intervention with other quality and improvement initiatives
CCG Pharm	A, B, C	CCG based pharmacist	Overview of medicines optimisation activities across the Clinical commissioning group Implementing intervention in three different practices
GP1	D	GP	Prescribing lead for practice
GP2	E	GP	Prescribing lead for practice
GPAdmin1	E	GP Admin- Booking clerk	Administered recall system for patients requiring monitoring
PM (Joint interview with PN)	G	GP- Practice manager	Overview of medicines safety and quality and improvement initiatives for the practice
PN (Joint interview with PM)	G	GP—Practice Nurse	Involved in quality and improvement initiatives for the practice
GP6	H	GP	No direct involvement with dashboard—communicated with pharmacist
GP7	H	GP	No direct involvement with dashboard—communicated with pharmacist
P1	F	Practice based pharmacist	Employed by practice—intervention only part of role
P2	G	Practice based pharmacist	Employed by practice—intervention only part of role
P3	E	Practice based pharmacist	Employed for the specifically to implement the intervention
P4	D, H	Practice based pharmacist	Employed by practice intervention only part of role
P5	B, E, I	Practice based pharmacist	Employed for the specifically to implement the intervention
P6	J, K, L, M, N, O	Practice based pharmacist	Neighbourhood Integrated Practice Pharmacist
P7	H	Practice based pharmacist with a prescribing qualification	Employed by practice—intervention only part of role
P8	G	Practice based pharmacist	Employed by practice—intervention only part of role
P9	J, K, L, M, N,	Practice based pharmacist	Neighbourhood Integrated Practice Pharmacist
P10	Q, R, S, T	Practice based pharmacist	Neighbourhood Integrated Practice Pharmacist
P11	N, O	Practice based pharmacist	Neighbourhood Integrated Practice Pharmacist
P12	P	Practice based pharmacist	Employed by practice—intervention only part of role

Key: CCG–Clinical Commissioning Group; GP–General Practitioner; P–Pharmacist; PM–Practice Manager; PN–Practice Nurse

### Ethics approval and consent to participate

Ethical approval for the study was granted by the North West–Greater Manchester East NHS Research Ethics Committee (reference15/NW/0792). All interview participants gave written informed consent to take part in the study, and for the interviews to be audio recorded and transcribed verbatim.

### Data collection

The interview schedules (S2 Appendix) were informed by NPT [35–38] to examine how participants made sense of the intervention, the interactional work involved in its adoption and implementation, the ways the intervention was used in practice and how it was appraised. Follow up interviews were conducted with four participants opportunistically sampled from those who were working in practices that were early implementers of the intervention and sampled to reflect the range of different professionals using the dashboard. The follow-up interviews were designed to explore changes in the intervention over time. Interviews were conducted by MJ at the general practice where the participant was working, on university premises, at the local NHS hospital trust or CCG offices. The first interviews were conducted between March and December 2016 as the intervention was implemented in different practices across Salford. Follow-up interviews were conducted between July and September 2016. Participants were reimbursed with £20 shopping vouchers per interview.

### Data analysis

MJ led the data analysis, which was thematic and conducted concurrently with data collection [41]. After the first six interviews were conducted the transcripts were read independently and discussed by MJ, RNK and DLP to uncover emerging themes [41]. These formed an initial thematic coding framework, and were applied to the transcripts by MJ using QSR NVivo 11 in order to manage the data. As data collection continued, further transcripts were added to the dataset and coded alongside the initial interviews and the thematic coding framework developed and changed. Data collection continued until, it was agreed in discussions between MJ, DLP and RNK that no new codes or themes were emerging from the interviews and thus data saturation had been reached. The coding and emerging themes were discussed by MJ, DLP and RNK and consensus reached. Consistent with NPT these themes were then grouped, mapped and interpreted alongside the four NPT constructs [42].

## Results

Twenty-one interviews were undertaken with twenty–two participants: three CCG managers, twelve pharmacists (one of whom was qualified to prescribe medicines independently), four GPs, one practice nurse, one practice administrator and one practice manager to explore a range of different views of those exposed to the intervention (see Table 2) between April and December 2016. Four individual staff from these eighteen practices declined to take part mainly due to workload and time issues. Additionally two CCG managers declined to take part.

Interviews lasted between 14 and 62 minutes. Each participant was interviewed once individually, with the exception of a practice nurse and practice manager who took part in a joint interview. Three months later, a further four interviews were conducted with a purposive sample of the participants who had taken part in early interviews (three pharmacists and one GP) to explore whether any changes had occurred. The main findings are mapped onto the four NPT constructs as summarised in Table 3.

**Table 3:** Normalisation process theory constructs mapped to the identified themes.

**COHERENCE. Sense-making work: How pharmacists and GP staff understood and attributed worth to the intervention.**	**COGNITIVE PARTICIPATION. Relational work that people did to set up and establish the intervention; enrolment, engagement and buy-in**
The dashboard was perceived as easy to use Pharmacists considered the dashboard added value to their work The intervention was perceived to be embedded in the context of wider medicines safety activities. Pharmacists were largely responsible for **integrating it into the GP practice.** Pharmacists considered that they overcame **resistance to the intervention** through sense-making work with other practice staff.	Collaborations established the intervention with pharmacists leading this by demonstrating the dashboard to others within the practice. There was varied access and engagement from different stakeholders Pharmacists felt that **trust and confidence** in the dashboard and in the work of the pharmacists highlighting hazardous prescribing and initiating changes to patient’s medication was important in establishing the intervention.
**COLLECTIVE ACTION. The work undertaken to sustain and operationalise the intervention: interactions, relationships and collaborations.**	**REFLEXIVE MONITORING. Appraising and monitoring: how pharmacists and clinicians reflected upon and appraised the intervention**
**Communication and collaboration** was seen as important Collaborations between pharmacists and prescribers were based upon **agreement and planning**. The dashboard was perceived to help to build relationships.	Pharmacists met together and gave feedback to the research team which led to **developments in the dashboard.** The intervention was seen as a tool that could lead to changes in work practices Pharmacists broadened their actions from attending to high risk prescribing cases to undertaking full medicine reviews with patients. Education and development important to the intervention The dashboard was seen as presenting opportunities for **further medicines safety activities.**

### Coherence: Making sense of, and setting up, the intervention in the context of pharmacist and GP working practices

#### Perceptions of ease of use

Pharmacists, GP staff and CCG managers felt the intervention to be valuable: it provided quick and easy access to actionable data and streamlined the way they worked. The dashboard potentially saved time since it provided near real-time information on hazardous prescribing. Compared to similar initiatives it did not require laborious audits to be run in the GP clinical system by practice staff:

*"I think the main benefit is that it’s just how quick and easy it is to access these patients. […] Running the searches on [the GP clinical system] I think is a nightmare*. *[…] For me I literally go in, in the morning […] and I have all my patients straightaway” (Practice pharmacist -P11)*

And similarly acknowledged by other practice pharmacists;

*“…it’s just quick and easy isn’t it? You can turn up at a surgery, log on the dashboard, ‘cause you’ll have access to that surgery, and within an hour you could have made several safety interventions, from just turning up at a random doctor’s surgery, […]you can just walk in*, *and you could have made quite an impact.” (Practice Pharmacist -P3)*

#### Pharmacists considered the dashboard added value to their work

Engagement with the dashboard enabled pharmacists to review a patient's medical records and undertake actions to ensure they were no longer at risk. This involved a process of sense-making in which the pharmacists considered the dashboard added value to their work, which in turn motivated them to continue to use it:

*“At the moment there’s seven […] patients that have fallen off [*i.e. no longer highlighted by the dashboard as at risk] *in the time that I’ve been there that I know that I have personally reviewed*. *They’re safer now*. *[…] For it to be quantifiable like that*, *is really nice” (Practice Pharmacist -P9)*

#### Wider medicine safety activities

The intervention was also understood in the context of wider medicine safety activities. Pharmacists and clinicians were more likely to use the dashboard if it helped to fulfil the requirements of other incentivised schemes. General practices were described as busy and under pressure from competing priorities such as the constant flow of new initiatives.

*“…from what I understand in GP land*, *they get …every year they get something new thrown at them, something new, something exciting, something that is basically going to change the way they get paid.” (Practice Pharmacist -P2)*

#### Integrating the intervention into practices

Pharmacists worked with GPs and other practice staff to build an understanding of the intervention and what it could achieve. This often involved the pharmacists demonstrating the dashboard to clinicians and other staff members, as individuals or small groups, in order to integrate the intervention into the practices.

*“So*, *showed them* [the GP] *the dashboard*, *where I had got the information from*, *because it was a good way to get his buy-in into the dashboard*, *to say*, *oh*, *look what this can do*, *look what it has found*. *(Practice Pharmacist -P1)*

#### Resistance to the intervention

There was some resistance from clinicians and GP staff, and a need to build awareness that the intervention could "*save (medication safety) problems in the future" (Practice Pharmacist -P11)*. Similarly one pharmacist commented that potential resistance from GPs was related to a perception that the intervention would involve extra work, and this first had to be overcome in order for any benefits to be realised.

### Cognitive participation: Enrolment and engagement to establish the intervention

#### Collaboration established the intervention

Collaboration between stakeholders was important for establishing the intervention. Giving clinicians access to the dashboard and showing them the tool was perceived as important to get practice staff “*on board”*.

*“So I got involved in a lot of the setup in the practices that I’ll be working in and also showing them [practice staff] the dummy [version of the dashboard]*, *and then getting them on board with it. And the initial feedback we got was all very positive. A lot of the GPs are on board. The practice manager is completely on board and excited for it. And […] most of them have access now as well.” (Practice Pharmacist—P9)*

Clinicians provided a way of linking the work of the pharmacist to the wider prescribing activities in the practice. Pharmacists spoke of needing *“a clinician to go to regularly” (Practice Pharmacist -P2)* partly because prescribing changes could only be carried out by a clinician.

#### Varied access and engagement from different stakeholders

The intervention was perceived to be owned by the pharmacists, as pharmacist P5 reflected:

*"I don’t know if any of them [GPs] are looking at the dashboard themselves*. *I think the dashboard is seen as my thing. […] We’ve got them set up with log-ins but I think it could be that they don’t really know how to use it” (Practice Pharmacist -P5)*

A lack of involvement from clinicians was legitimised by suggestions that pharmacists were experts in medicines with the appropriate skill-sets, and consequently the ideal people to use the system. It was assumed that pharmacists were much more likely to engage with the system than clinicians because "*they know what they’re looking for and what they’ve already actioned and what still needs actioning*,*" (Practice Manager -PM)*.

#### Trust and confidence

The intervention helped to build respect, improve trust and develop relationships between pharmacists and GPs. Pharmacists saw the dashboard component as offering opportunities to demonstrate their skills and to further develop their role working within general practice settings. In setting up the intervention it was important that clinicians had trust and confidence in both the dashboard and the work of the pharmacist. Such trust and confidence could be achieved as one pharmacist reflected by them *"proving [their] worth" (Practice Pharmacist -P1)* through their work in helping to resolve hazardous prescribing cases and making changes made to patient's medication. This added value to the role of a practice pharmacist allowing the pharmacists to develop their professional role in general practice. Trust and confidence in the intervention was also achieved in that the feedback provided by the dashboard was seen to be depersonalised:

*“Yeah, it does [improve things in practice] and having this tool depersonalises [feedback]*, *because it is …this system has picked up that you have prescribed this. It’s not …you know, you’ve done this and I don’t think it’s safe …it’s the system has picked this up, so it depersonalises everything […]so it’s a good way of getting feedback without making it personal.” (GP-1)*

### Collective action: Work undertaken to adopt and sustain the intervention including communication, collaborations and divisions of labour

#### Communication and collaboration

Communication and collaboration were seen as important for the intervention and for embedding the intervention into routine practice. The communication of messages regarding patients highlighted by the dashboard as being at risk of potentially hazardous prescribing practice was primarily initiated and led by the pharmacists. This was important work in order to establish how the intervention was going to be adopted and operationalised in the practices. Pharmacists adopted different ways of communication with clinicians, in order to engage them with the intervention. Since many of the pharmacists had worked in hospital settings previously this involved contrasting their previous ways of working to the different context of primary care, as well as building an understanding of the complexities of primary care where group meetings were perceived as difficult:

*"It’s difficult, […] when’s the best time to approach doctors to discuss things*, *[…] when the surgery is not on, they’re on home visits or they’re in meetings, it’s quite a different way of working. So that’s probably one barrier […], so it’d be difficult probably to get everybody together unless you went to the practice meeting on another day. “(Practice Pharmacist -P3)*

Most pharmacists relied upon clinicians to make changes to patients’ medication. One CCG manager, who also worked as a pharmacist, talked of providing clinicians with "*simple messages"* if needed and described communication as only driven from necessity.

*“that’s how I’ve been using it […], not bothering the GP too much if they don’t need to be*, *[…] just simple messages and where patients do need acting on, just passing the NHS number to the GP and getting that acted upon.”(CCG Pharmacist–CCG Pharm)*

#### Agreement and planning

The collaboration between pharmacists and clinicians in delivering the intervention was characterised by agreement and planning. Pharmacists discussed how they worked with GPs *"to have agreed an action plan" (Practice Pharmacist -P10)*. The pharmacist worked through lists of patients potentially affected by hazardous prescribing but *“action plans”* were sent to the relevant clinician. There was therefore some division of labour happening; pharmacists undertook specific tasks in highlighting patients at risk and suggesting a suitable plan of action but then these would often be dealt with by clinicians who *“would decide whether to contact the patient*, *whether to action it or not” (GP1)*. This role allocation did have the benefit of giving a sense of value to the intervention. Pharmacists valued the dashboard in that it was perceived to potentially "*lead on to making general practitioners confident in what pharmacists can do within general practice" (Practice Pharmacist -P10)*. GPs valued the additional presence of the pharmacist since it was seen to ensure “…*things don’t get missed…” (GP7)*. And as this pharmacist reflected;

*“Well that’s the benefit of having it pharmacist led*, *isn’t it that you know somebody is going to follow it up and make sure it’s done and keep checking and keep going back and, you know. Sometimes you give GPs work and they’re just so overloaded that it’s just another thing for them to be looking at and to be doing …” (Practice Pharmacist -P12)*

#### Building relationships

It was also seen as important that the pharmacist developed and built relationships with practices in order to make the intervention more successful. Relationships between the pharmacist and others were perceived to be built through the use of the dashboard;

*“So*, *I am using it as an exercise, really, in way of building a relationship with the GPs as well and for them to see what the SMASH dashboard can do for them. So, I know, potentially, not everyone is using …taking that approach, but I just think it, one, gets SMASH, the dashboard some PR and is also, yeah, helping me to build some relationships.”(Practice Pharmacist -P10)*

### Reflexive monitoring: How pharmacists and clinicians reflected upon and appraised the intervention and the potential for sustaining long-term system change

#### Developments in the dashboard

The pharmacists met regularly in a process of sharing good practice and developing different approaches to working with the dashboard and within their practices. One such meeting led to a change to the dashboard in the development of a ‘note feature’ that allowed users to give reasons for the actions that had been taken in response to managing patients that had been highlighted as at risk of hazardous prescribing.

*“The fact that now you’ve added the notes system*, *so I can flag up if we have reviewed somebody, just to avoid duplication, works good.” (Practice Pharmacist -P6)*

#### Changes in work practices

The intervention was seen as a tool that could develop learning and lead to changes in work practices and the quality and safety of patient care, for instance in making changes in the way recall letters for blood test monitoring were conducted.

*“One of the indicators is the Amiodarone and people coming in for six monthly bloods*, *so I’d worked with her to put… a recall and it’s just to put it on the system and then she checks the recalls on a monthly basis, […] so she can put it on so that every six months they will come up in this recall search […] then we got everybody on Amiodarone in general and then she put a recall on for me for all those people so that nobody ever will breach that indicator again”. (Practice Pharmacist -P3)*

#### Pharmacist broadening their actions

The role of the pharmacist within the intervention was to engage in supporting these activities within the practices but in some practices this was seen as opportunity to translate the evidence provided by the dashboard into transformations in care by conducting a more comprehensive medication review for patients:

*“We’ve actually broadened the remit a little bit*, *because […]when you have a patient with one thing […] something that’s identified on the dashboard, there often may be other things, and our view is holistic care. […]When we look at those patients, we’re obviously looking at the indicator that flags, but also making sure we look at the wider patient as well.” (Practice Pharmacist -P6)*.

#### Education and development

Several respondents discussed education for individual clinicians, and it was acknowledged that working on high risk prescribing needed to lead to education in order to maintain the quality of prescribing. GPs wanted to be *“told if we are prescribing unsafely” (GP1)*, and would use the dashboard for their *“own education” (GP1)*. Pharmacists spoke of the need for education and awareness as part of “*a personal development as well*” *(P4)* and for the need for clinicians to “*take more of an ownership” (Practice Pharmacist -P3)* in order to bring about change and to “*sustain the difference*” *(Practice Pharmacist -P2)*. Education of clinicians was often initiated by pharmacists and involved changes in the interactions between pharmacists and clinicians, in terms of the ways they communicated messages, and feedback to prescribers from pharmacists that could result in embedding the intervention into routine practice and in learning to avoid situations of hazardous prescribing when managing other patients.

#### Future medicines safety activities

The intervention was also seen as presenting opportunities for the further quality and medicines safety programmes in the future. One CCG manager reflected that;

*“I think it’ll give us a useful tool to be able to perhaps design our programmes of work*, *and also thinking about if we’re going to run any quality programmes in the future, it will hopefully help us to design what we’re working on because it will give us that information, give us that baseline that we need so often.” (CCG1)*

Using NPT provided therefore valuable insights into the ways in which developing relationships, collaborations and communication between health professionals could lead to the implementation, adoption and sustainability of the intervention. The results are summarised in relationship to the NPT constructs in Table 3. This highlights the main themes under each construct.

## Discussion

This study explored the ways in which a complex, pharmacist-led intervention, using near real-time electronic audit and feedback, was implemented, adopted, and embedded into everyday practice creating a learning health system to improve medication safety in primary care. One of the key elements of a rapid learning health system is for clinicians to have access to data and searchable health records at a local level.[23, 43] The intervention allowed for a rapid learning health system to evolve through the connection of the real time data in the dashboard to changes in patient’s medication. In this the role of the practice based pharmacist was pivotal. Good collaborative relationships between the pharmacists and other health professionals was important in making sense of the intervention, engaging and implementing it and sustaining it in everyday practice. In a systematic review by Lainer et al IT interventions for were seen as successful for improving medication safety in primary care if the intervention involved collaboration between pharmacists and clinicians. [19]

The pharmacists engaged with the dashboard more than general practitioners and it was perceived as being owned by the pharmacists. Studies of earlier interventions to improve prescribing in primary care have reported variations in engagement.[39, 40] and have seen differences in roles with some health professionals utilising technology and others rejecting it.[44] In the present study there were positive outcomes from the differences in roles since the intervention allowed pharmacists to demonstrate their professional skills through the use of the system and develop the role of clinical pharmacy in general practice. This runs somewhat counter to examples in previous literature that has seen some primary care IT systems reduce the autonomy and professional discretion of health professionals.[13, 45] It may well suggest that when technology does fit with the professional norms and values of a specific group of health professionals it is more likely to be accepted.

### Strengths and limitations

The NPT constructs drew out the multifaceted nature of the intervention which included the dashboard itself, the negotiation of user roles, the fitting of the intervention into general practice, the wider considerations of the CCG and the pharmacist role. The collective action construct was able to reveal how the dashboard meant different things to different people: pharmacists using it to feedback instances of high risk prescribing, and GPs using it for their own education. This fits with an extended version of NPT that focuses upon the complexity of contexts, the changes in roles and plasticity of the intervention. [46] Using NPT was particularly useful in unpicking what McEvoy et al., have described as the *“implementation journey”* where the gradual processes of the intervention towards its adoption in everyday practice is understood.[47] An important limitation to this study was that the evaluation team also developed the intervention. Whilst this allowed for some changes to be made to the intervention it potentially had implications for the ways in which we assumed the dashboard would be utilised in practice. This may have an impact upon the transferability of these findings to other settings. Furthermore transferability may be limited by the specific nature of the intervention setting and particularly the roles of practice pharmacists as this was an innovative pharmacist-led intervention. A further limitation is the limited number of follow-up interviews. As described above this was for pragmatic reasons based upon the timelines of the evaluation which did not facilitate further interviews to be undertaken. It would therefore be beneficial for future research to evaluate the roll-out of the intervention in wider settings. As a purely qualitative study we could not evaluate the impact of the intervention. The research team are, however collecting quantitative data and this will be reported in the future.

### Implications for improving medication safety as a learning health system

Audit and feedback interventions using dashboards and prescribing safety indicators have a utility in primary care medication safety and could be further implemented across primary care. A LHS infrastructure that allows for real-time electronic audit and feedback data to be available to clinicians at the point of care has the potential to translate evidence swiftly into practice.[22, 23, 48] Our findings demonstrate that such a rapid learning health system can help connect data to changes in medication safety particularly through the role of pharmacists. Such interventions thus, as Friedman has suggested,[25] lead to continuous cycles of improvement since the real time data in the dashboard is continually updated. In this way, prescribing behaviours and patterns can be reflected upon and changed; new prescribing will be changed in the dashboard and new learning facilitated. This closed loop enables users to see the results of their own actions reflected in the dashboard and thus provide positive feedback. Central to the intervention was the connection between the dashboard and the clinical pharmacist and the collaborative processes involved in its use and sustainability. The PINCER study [21] highlighted the importance of the communication between pharmacists and clinicians. The present study builds upon that by seeing the importance of having the pharmacist embedded within practices which led to a more active relationship with clinicians based upon agreement and planning. In the future, similar interventions might take into consideration the importance of these collaborative processes, how technology use can be translated into practice and how rapid learning health systems need to fit into existing work practices.[24, 35] The findings of this study are of particular relevance given the national implementation of clinical pharmacists in general practices led by NHS England [49] Internationally it has been suggested that pharmacists can reduce GP workload and work pressure and deliver a range of interventions[50–53] Further examination of the professional identity of this role and how it is evolving within primary care particularly given the WHO Global Challenge to improve medication safety [54] would be beneficial.

## Conclusions

Medicine optimisation activities in primary care may be enhanced by the implementation of a clinical pharmacist-led electronic audit and feedback system that highlights potentially hazardous prescribing in general practices. Such an intervention enables a rapid learning health system that swiftly translates data from electronic health records into changes in practice to improve patient safety. At the centre of this learning health system, and pivotal to its success and sustainability, was the role of the clinical pharmacist who provided the link between data and changes in care. Our study thus highlighted the importance of the combined use of information technology and the emerging and developing role of clinical pharmacists in general practice The use of NPT as an analytical framework provided valuable insights into the work of implementation, adoption and sustainable execution of the intervention, revealing that it was critically dependent upon interactions between the pharmacist, clinicians and other staff members.

## Supporting information

S1 Checklist COREQ research checklist. (PDF)

S1 Appendix Prescribing safety indicators. (PDF)

S2 Appendix Interview schedule. (PDF)
