# Supplementary material for: Developing a learning health system: Insights from a qualitative process evaluation of a pharmacist-led electronic audit and feedback intervention to improve medication safety in primary care
Source: PLoS One. 2018 Oct 26;13(10):e0205419. doi: 10.1371/journal.pone.0205419 (PMC6203246; doi:10.1371/journal.pone.0205419)
Supplement: S1 Checklist — (PDF) [file pone.0205419.s001.pdf]

Developing a learning health system: insights from a pharmacist-led electronic audit and feedback intervention to improve medication safety in primary care

COREQ checklist

Note: in order to minimize the length of the manuscript, some of the details on the checklist (marked '*') are not included in the manuscript.

Guide question		*Response*	*Page number in manuscript*
1	Interviewer/facilitator	MJ conducted the interviews.	P10
2	Credentials	MJ holds an PhD in Pharmacy Practice	*
3	Occupation	MJ : Research Associate in medication safety;	*
4	Gender	MJ male	*
5	Experience and training	MJ has previous experience of undertaking qualitative research in healthcare at PhD and postdoctoral level	*
6	Relationship established	The researchers were not known to the participants prior to the study	*
7	Participant knowledge of the researcher	Participants were made aware of the reasons for doing the research via the information which was sent to the participant prior to the interview	Ethics statement P9
8	Researcher characteristics	The researchers had identified the study topic as part of larger programmes of work in their research groups, medication safety in primary care.	*
9	Methodological orientation and theory	Normalisation process theory. The analysis was thematic.	P7-8, P10
10	Sampling	The sampling frame was GP staff and pharmacists in practices where the intervention was introduced, and CCG staff who had responsibilities for medicines optimisation and quality improvement. Individual potential participants were directly contacted by MJ via telephone or email and invited to take part in an interview. Participants were recruited from 18 practices purposefully reflecting a range of different contexts which may have had an impact upon how the intervention was implemented and adopted. These included, variations in practice size, social deprivation and clinical systems used (EMIS Web; InPractice systems VISION).	P8-9
11	Method of approach	Participants were approached by telephone or email	P9
12	Sample size	22 participants	Results section P11, Table 2 P11
13	Non-participation	A number of possible participants were approached but declined to participate. Predominantly this was for reasons of time, workload or lack of use of the system.	P11
14	Setting of data collection	Interviews were conducted by MJ at the general practice where the participant was working, on university	P10

		premises, at the local NHS hospital trust or CCG offices.	
15	Presence of non-participants	No non-participants were present	*
16	Description of sample	See Table 2 of the main manuscript	P11
17	Interview guide	The interview schedule was informed by NPT to examine how participants made sense of the intervention, the interactional work involved in its adoption and implementation, the ways the intervention was used in practice and how it was appraised	P9-10
18	Repeat interviews	Follow up interviews were conducted with a sample of participants to explore changes in the intervention	P12
19	Audio/visual recording	Audio recording only, with consent from the participant	*
20	Field notes	None	*
21	Duration	The interviews lasted between 14 and 62 mins.	P12
22	Data saturation	Data collection continued until saturation was reached and no new themes emerged from the interviews and focus groups.	*
23	Transcripts returned	No transcripts were returned to participants	*
24	Number of data coders	MJ coded the data but regular discussions codes were held with all DLP and RNK	P10
25	Description of the coding tree	A coding tree description is not given. Coding themes were developed from the first transcripts and then once developed mapped onto NPT constructs	P10
26	Derivation of themes	Emerging thematic codes were applied to the data and new themes emerged from the data. This is described in the analysis section	P10
27	Software	QSRNvivo 11 software was utilised to manage the data	P10
28	Participant checking	No	*
29	Quotations presented	Please see the results section of the manuscript	P10-P23
30	Data and findings consistent		P10-P23
31	Clarity of major themes		P10-P23
32	Clarity of minor themes		P10-P23
