# Supplementary material for: Developing a learning health system: Insights from a qualitative process evaluation of a pharmacist-led electronic audit and feedback intervention to improve medication safety in primary care
Source: PLoS One. 2018 Oct 26;13(10):e0205419. doi: 10.1371/journal.pone.0205419 (PMC6203246; doi:10.1371/journal.pone.0205419)
Supplement: S1 Appendix — (PDF) [file pone.0205419.s002.pdf]

Salford Medication Safety Dashboard - Prescribing Safety Indicators

**Description of indicator**	**Eligible patients at risk (denominator)**	**Patients flagged by system (numerator)**
Prescription of an oral NSAID without co-prescription of an ulcer-healing drug in a patient aged ≥65 years.	Patients aged ≥ 65 years on the audit date without prescription of an ulcer-healing drug within the 3 months leading up to the audit date.	Patients prescribed at least one oral NSAID within the 3 months leading up to the audit date.
Prescription of an oral NSAID without co-prescription of an ulcer-healing drug to a patient with a history of peptic ulceration.	Patients aged ≥ 18 years on the audit date with a history of peptic ulceration at least 3 months before the audit date without co-prescription of an ulcer-healing drug within the 3 months leading up to the audit date.	Patients prescribed an oral NSAID within the 3 months leading up to the audit date.
Prescription of an antiplatelet drug without co-prescription of an ulcer-healing drug[5] to a patient with a history of peptic ulceration.	Patients aged ≥ 18 years on the audit date with a history of peptic ulceration at least 3 months before the audit without co-prescription of an ulcer-healing drug within the 3 months leading up to the audit date.	Patients prescribed an antiplatelet drug within the 3 months leading up to the audit date.
Prescription of warfarin or NOAC in combination with an oral NSAID.	Patients aged ≥ 18 years on the audit date prescribed warfarin or NOAC within the 3 months leading up to the audit date.	Patients prescribed an oral NSAID within the 3 months leading up to the audit date.
Prescription of warfarin or NOAC in combination with and an antiplatelet drug without co-prescription of an ulcer-healing drug.	Patients aged ≥ 18 years on the audit date prescribed warfarin or NOAC within the 3 months leading up to the audit date without co-prescription of an ulcer-healing drug within the 3 months leading up to the audit date.	Patients prescribed an antiplatelet drug within the 3 months leading up to the audit date and within 28 days of the prescription for Warfarin or NOAC
Prescription of aspirin in combination with another antiplatelet drug without co-prescription of an ulcer-healing drug.	Patients aged ≥ 18 years on the audit date prescribed aspirin within the 3 months leading up to the audit date without co-prescription of an ulcer-healing drug within the 3 months leading up to the audit date.	Patients prescribed another antiplatelet drug within the 3 months leading up to the audit date and with 28 days of the prescription for aspirin.
Prescription of a non-selective beta-blocker to a patient with asthma.	Patients aged ≥ 18 on the audit date with a Read code for asthma at least 3 months before the audit date and no Asthma resolved code.	Patients prescribed a non-selective beta-blocker within the 3 months leading up to the audit date.

Prescription of a long-acting beta-2 agonist inhaler (excluding combination products with inhaled corticosteroid) to a patient with asthma who is not also prescribed an inhaled corticosteroid.	Patients aged ≥ 18 on the audit date with a Read code for asthma at least 3 months before the audit date and no Asthma resolved code, who have been prescribed a long-acting beta-2 agonist inhaler (excluding combination products with inhaled corticosteroid) within the 3 months leading up to the audit date.	Patients who have not been prescribed an inhaled corticosteroid within the 3 months leading up to the audit date.
Prescription of an oral NSAID to a patient with heart failure.	Patients aged ≥ 18 on the audit date with a Read code for heart failure at least 3 months before the audit date.	Patients prescribed an oral NSAID within the 3 months leading up to the audit date.
Prescription of an oral NSAID to a patient with chronic renal failure (eGFR <45)	Patients aged ≥ 18 on the audit date an eGFR <45 at least 3 months before the audit date.	Patients prescribed an oral NSAID within the 3 months leading up to the audit date.
Prescription of an ACE inhibitor, loop diuretic and oral NSAID to a patient with chronic renal failure (eGFR < 45)	Patients with chronic kidney disease stage 3B, 4, or 5 (or eGFR < 45) and prescribed ACEI and loop diuretic	Patients prescribed an oral NSAID within 3 months leading up to audit date
Missing thyroid function test in the past 6 months/patients receiving repeat amiodarone	Patients prescribed amiodarone 6-12 months before audit date and again within 6 months leading up to audit date	Patients who have NOT had thyroid function test in 6 months leading up to audit date
Missing full blood count or liver function test in the past 3 months/patients receiving repeat methotrexate	Patients prescribed methotrexate 3-6 months before audit date and again within 3 months leading up to audit date	Patients who have NOT had liver function test or full blood count in 3 months leading up to audit date
NOAC - New oral anticoagulant drug<br>NSAID- Non-steroidal anti-inflammatory drug NSAID- Non-steroidal anti-inflammatory drug<br>eGFR- Estimated Glomerular Filtration Rate.<br>ACEI - Angiotensin Converting Enzyme Inhibitor		
