# Supplementary material for: Developing a learning health system: Insights from a qualitative process evaluation of a pharmacist-led electronic audit and feedback intervention to improve medication safety in primary care
Source: PLoS One. 2018 Oct 26;13(10):e0205419. doi: 10.1371/journal.pone.0205419 (PMC6203246; doi:10.1371/journal.pone.0205419)
Supplement: S2 Appendix — (PDF) [file pone.0205419.s003.pdf]

INTERVIEW SCHEDULE – GP Staff (First Interview)

Interviewer introduction:

I am [….] and I am a researcher at the University of Manchester. We are carrying out a project looking at the use of a medication safety dashboard and clinical pharmacist-led education and feedback in Salford. We would like to find out more about the views of healthcare professionals who have worked with this system.

This interview will last for approximately forty minutes, and during that time I'd like to discuss your experience of using the electronic dashboard and engaging with the clinical pharmacist. I should remind you that the interviews are confidential and will be used only for our own research. I'd like to record the discussion if that is okay with you; this is simply to help me capture all of the information that comes out of it. Before we begin, I'd like to provide some ground rules for the discussion:

- You are being digitally recorded, so speak clearly;
- We will anonymise the transcript so that nobody can be identified by name. However, please try to avoid naming specific healthcare professionals or locations;
- Everything discussed here is confidential. However, if you were to reveal anything that would place you at somebody else at risk of harm, we may have to report this to a clinical supervisor or manager.

Unless you have any questions for me, then we can begin.

Interview questions: (supplementary questions)

1. What is your experience of working with the medication safety dashboard?
2. What were your expectations of the intervention (the dashboard and the work of the pharmacist)? (What was your understanding of it?)
3. Can you describe how the intervention has worked in your practice? (Who has used the dashboard? What interactions have there been with the pharmacist? Why have those people been involved?)
4. What particular things have helped make the intervention work in your practice?
5. What things have meant that the intervention has not worked in your practice?
6. How, if at all, have things changed from the way you worked before? (What are those changes? What adjustments have been made to work practices? Have there been any role changes? Are things done differently? Why have those changes been made?)
7. Is there anything else that you think should be discussed given our research topic?

INTERVIEW SCHEDULE – GP Staff (Second Interview)

Interviewer introduction:

I am [….] and I am a researcher at the University of Manchester. We are carrying out a project looking at the use of a medication safety dashboard and clinical pharmacist-led education and feedback in Salford. We would like to find out more about the views of healthcare professionals who have worked with this system.

This interview will last for approximately twenty minutes, and during that time I'd like to discuss your experience of using the dashboard and engaging with the clinical pharmacist. I should remind you that the interviews are confidential and will be used only for our own research. I'd like to record the discussion if that is okay with you; this is simply to help me capture all of the information that comes out of it. Before we begin, I'd like to provide some ground rules for the discussion:

- You are being digitally recorded, so speak clearly;
- We will anonymise the transcript so that nobody can be identified by name. However, please try to avoid naming specific healthcare professionals or locations;
- Everything discussed here is confidential. However, if you were to reveal anything that would place you at somebody else at risk of harm, we may have to report this to a clinical supervisor or manager.

Unless you have any questions for me, then we can begin.

Interview questions: (supplementary questions)

1. Can you describe how the intervention worked in your practice? (Who used the dashboard? What interactions were there with the pharmacist? Why were those people involved?)
2. What particular things helped make the intervention work in your practice?
3. What things meant that the intervention did not work in your practice?
4. What was the impact of the pharmacist no longer visiting your practice?
5. How have things changed, if at all, in the way you work now because of the intervention? (What are those changes? What adjustments have been made to work practices? Have there been any role changes? Are things done differently? Why have those changes been made?)
6. Is there anything else that you think should be discussed given our research topic?

INTERVIEW SCHEDULE – Clinical Pharmacist

Interviewer introduction:

I am [….] and I am a researcher at the University of Manchester. We are carrying out a project looking at the use of a medication safety dashboard in Salford. We would like to find out more about the views of healthcare professionals who have used this system.

This interview will last for approximately forty minutes, and during that time I'd like to discuss your experience of using the dashboard and engaging with general practice staff. I should remind you that the interviews are confidential and will be used only for our own research. I'd like to record the discussion if that is okay with you; this is simply to help me capture all of the information that comes out of it. Before we begin, I'd like to provide some ground rules for the discussion:

- You are being digitally recorded, so speak clearly;
- We will anonymise the transcript so that nobody can be identified by name. However, please try to avoid naming specific healthcare professionals or locations;
- Everything discussed here is confidential. However, if you were to reveal anything that would place you at somebody else at risk of harm, we may have to report this to a clinical supervisor.

Unless you have any questions for me, then we can begin.

Interview questions: (supplementary questions)

1. What is your experience of working with the medication safety dashboard?
2. Can you describe how the intervention has worked in this practice? (Who has used the dashboard? What interactions have there been between yourself and practice staff? Why have those people been involved?)
3. What particular things have helped make the intervention work in this practice?
4. What things have meant that the intervention has not worked in this practice?
5. Is there anything else that you think should be discussed given our research topic?

INTERVIEW SCHEDULE – CCG Pharmacist or Manager

Interviewer introduction:

I am [….] and I am a researcher at the University of Manchester. We are carrying out a project looking at the use of a medication safety dashboard in Salford. We would like to find out more about the views of healthcare professionals and managers who have used this system.

This interview will last for approximately forty minutes, and during that time I'd like to discuss your experience of using the dashboard and engaging with pharmacists and general practice staff. I should remind you that the interviews are confidential and will be used only for our own research. I'd like to record the discussion if that is okay with you; this is simply to help me capture all of the information that comes out of it. Before we begin, I'd like to provide some ground rules for the discussion:

- You are being digitally recorded, so speak clearly;
- We will anonymise the transcript so that nobody can be identified by name. However, please try to avoid naming specific healthcare professionals or locations;
- Everything discussed here is confidential. However, if you were to reveal anything that would place you at somebody else at risk of harm, we may have to report this to a clinical supervisor.

Unless you have any questions for me, then we can begin.

Interview questions: (supplementary questions)

6. What is your role?
7. How did you come to use the dashboard? (What were your expectations of it? What are your motivations to use it?)
8. What is your experience of working with the medication safety dashboard?
9. What benefits or disadvantages do you see in using the dashboard in your role? (What particular things have helped? What particularly things have not helped?)
10. What are the benefits or disadvantages of the dashboard to the CCG as a whole? (How do you see this in the future?)
11. Have you worked with others who are using it? (What interactions have there been between yourself and those people? Why have those people been involved?)
12. What is your understanding of how the dashboard will work in the GP practices? (How do you see the role of the clinical pharmacist as they go into the practices?)
13. Is there anything else that you think should be discussed given our research topic?
